# The Impact of Abduction Orthoses for the Treatment of Hip Dysplasia on the Development of Motor Skills: A Systematic Review and Meta-Analysis

**DOI:** 10.3390/jcm15041595

**Published:** 2026-02-18

**Authors:** Łukasz Pulik, Wiktor Kaczyński, Grzegorz Tomaszewski, Paweł Łęgosz

**Affiliations:** Department of Orthopedics and Traumatology, Medical University of Warsaw, Lindley 4 Str, 02-005 Warsaw, Polandpawel.legosz@wum.edu.pl (P.Ł.)

**Keywords:** developmental dysplasia of the hip, abduction orthoses, Pavlik harness, motor milestones

## Abstract

**Background:** Developmental dysplasia of the hip (DDH) is the most common musculoskeletal condition in infants, and it is routinely managed with abduction orthoses. Despite high treatment success rates, concerns persist regarding potential delays in motor milestone acquisition. This meta-analysis evaluates the impact of orthotic treatment in children with DDH on early motor development. **Methods:** PubMed, Web of Science, and Embase were screened from inception to 17 February 2025. The review followed PRISMA guidelines. Risk of bias was assessed using the ROBINS-I tool V2 and visualized with the ROBVIS application. Mean differences in motor milestone achievement timepoints were compared in months between the intervention and control groups using a random effects meta-analysis model. A meta-regression was conducted to explore potential moderators of effect size. The protocol was prospectively registered in PROSPERO (ID: CRD42025648186). **Results:** Four studies, including 952 children, were analyzed—335 were treated for DDH with abduction orthoses, and 617 were healthy controls. Pavlik harness was used in three studies (*n* = 235), while the Koszla brace was used in one study (*n* = 100). Children in the orthosis group began walking approximately 0.55 months later than healthy controls (95% CI: 0.40 to 0.70). Sitting was also delayed by 1.11 months (95% CI: 0.76 to 1.47). No significant difference was found for crawling. **Conclusions:** The use of abduction orthoses may result in a slight, clinically marginal delay in achieving motor milestones, such as sitting and unaided walking. However, given that untreated DDH can lead to severe functional limitations, early intervention with orthoses remains a justified and safe standard of care.

## 1. Introduction

Developmental dysplasia of the hip (DDH) is the most common musculoskeletal disorder in children, affecting 0.1–7.0% of the population, depending on ethnicity [1]. Several risk factors for DDH have been identified, including female sex, positive family history, breech presentation, and conditions associated with limited intrauterine space [1]. They refer to a spectrum of abnormalities involving insufficient development of the acetabulum and changes in proximal femoral bone anatomy. They lead to joint instability, subluxation, or complete dislocation of the hip. If left untreated, DDH can result in impaired gait, disability, and early-onset osteoarthritis [2].

Early diagnosis with ultrasound examination allows us to implement highly successful conservative treatment [3]. The use of orthosis is a common treatment for maintaining the hip in flexion and abduction, which is crucial for the development of the acetabulum [4]. Various orthoses have been used for the conservative treatment of developmental dysplasia of DDH in newborns, with the Pavlik harness and Tübingen brace being the most used today, while devices such as the Frejka pillow and Koszla orthosis were more prevalent in the past and are still used in some resource-limited settings. Treatment with abduction orthosis is generally continued until ultrasonographic maturity of the hip is achieved, which usually occurs within 3 to 4 months [5].

Treatment in modern types of orthoses is safe with respect to the risk of serious complications, such as avascular necrosis of the femoral head or femoral nerve palsy [6]. However, parental anxiety and psychosocial problems due to DDH on diagnosis and treatment have been reported [7]. Moreover, parents of patients often have concerns about the effects of orthoses and the acquisition of motor skills due to prolonged immobilization [8].

However, there is limited direct research on the impact of abduction orthosis treatment for DDH on the development of motor skills in infants [9,10]. Investigating the impact of abduction orthoses on motor milestone acquisition is crucial to address parental concerns and guide clinical practices. Clear evidence on whether orthoses delay sitting or walking would help optimize treatment protocols and improve communication with families.

The objective of this study is to evaluate whether the use of abduction orthoses in the treatment of DDH affects the timeline of motor development in children. Specifically, in this systematic review and meta-analysis, we aim to determine if children undergoing treatment for DDH with abduction orthoses achieve key developmental milestones, such as independent walking, sitting, or crawling, at a later age compared to their peers in a healthy population.

## 2. Materials and Methods

We conducted a search for original studies involving children treated for DDH using any form of abduction orthosis. We searched the following electronic bibliographic databases: EMBASE, PubMed, and Web of Science. The search only includes terms related to the topic of DDH treatment (keywords: developmental dysplasia of the hip; walking; abduction; orthosis). The database search was performed by the Medical University of Warsaw Library using predefined Boolean logic adapted to each platform. We searched the titles and abstracts published in English. The following operators were used: (‘developmental dysplasia of the hip’ OR ‘DDH’ OR ‘hip dysplasia’) AND (‘crawling’ OR ‘standing’ OR ‘walking’ OR ‘motor development’) AND (‘abduction’ OR ‘orthosis’ OR ‘treatment’ OR ‘brace’ OR ‘harness’ OR ‘pavlik’ OR ‘koszla’ OR ‘tubingen’ OR ‘tubinger’ OR ‘frejka’). The full search strategy was assigned a digital identifier (10.5281/zenodo.15831806).

**Figure 1:**
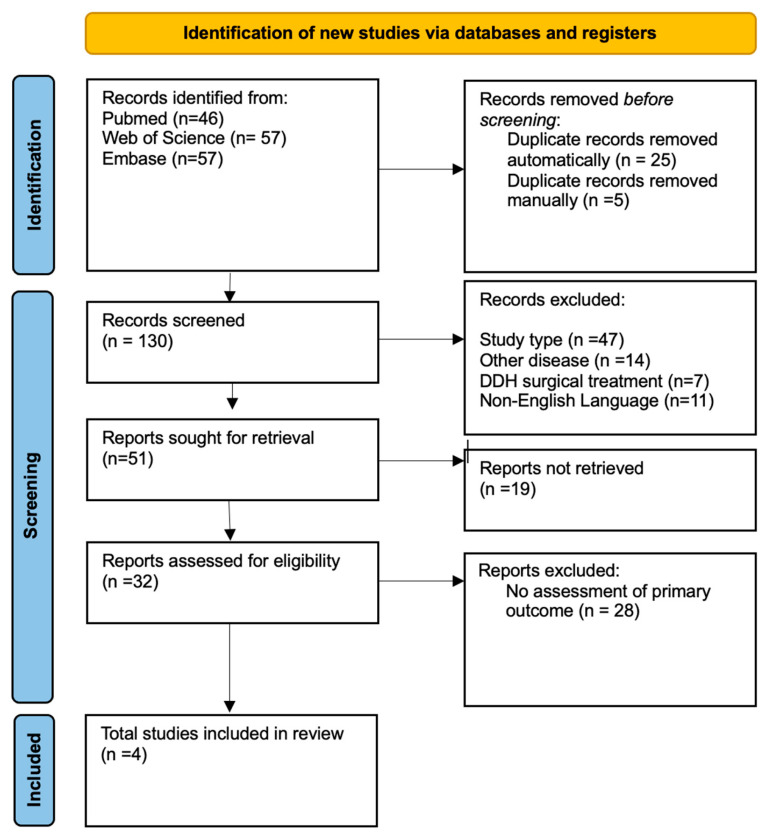
PRISMA flow diagram illustrating the study identification, screening, eligibility assessment, and inclusion process. After removal of duplicates and full-text evaluation, a total of four studies met the predefined eligibility criteria and were included in the qualitative and quantitative synthesis.

Screening proceeded in two stages: initial title and abstract review (Ł.P., W.K.), followed by full-text assessment of eligible studies (Ł.P., W.K.). Eligible study designs included cohort, longitudinal, retrospective, and prospective studies, with no restrictions on publication date (up to 17 February 2025). The decision to include studies regardless of publication year reflects the continued use of older orthotic devices, particularly in resource-constrained settings, despite the emergence of newer models that may offer improved safety profiles and potentially support more favorable motor development outcomes [11,12]. Therefore, excluding older devices such as the Koszla brace would risk limiting the external validity of the analysis and reducing its relevance to real-world clinical practice. We excluded review articles, meta-analyses, case reports, and case series. Studies with fewer than 10 participants were not included. Duplicate entries were removed using Endnote X9 (Clarivate Analytics, Philadelphia, PA, USA). The search results are reported in the PRISMA diagram (Figure 1).

Inclusion criteria were children treated for DDH using any type of abduction orthoses, with treatment initiated before 12 months of age. Exclusion criteria encompassed children requiring an abduction brace for reasons other than DDH, those treated surgically, those who underwent hip reduction under anesthesia, and those managed with a cast immobilization. The extracted data include gender, age, and population ethnicity; disease-related information, such as time of diagnosis; treatment-related information, including the type of orthosis, duration of treatment, age at treatment initiation, and complications; and outcome information, covering the onset of independent sitting, crawling, walking, and rolling.

The study primarily assessed the onset of independent walking as the key outcome, while secondary outcomes included the onset of sitting and crawling. A standard meta-analysis was conducted to pool effect sizes across studies. Effect estimates included mean differences (MDs) and 95% confidence intervals (CIs). Heterogeneity was assessed using Cochran’s Q-test and the I^2^ statistic. Where necessary, medians and interquartile ranges were converted to means and standard deviations (SDs) using the method proposed by Hozo et al. [13]. Meta-regression was performed to explore heterogeneity, pooled effect sizes, and potential sources of variation (duration of treatment, the timing of treatment initiation) [14]. Statistical analysis was performed using STATA (18.5 BE) (StataCorp LLC, College Station, TX, USA). The risk of bias in this systematic review was assessed using the ROBINS-I V2 tool. Two independent reviewers evaluated the studies, resolving any discrepancies through discussion. The results were visualized using the ROBVIS tool.

This study is a systematic review and meta-analysis of observational studies. The study is prospectively registered in the PROSPERO database (registration number: CRD42025648186) [15]. For manuscript preparation, the PRISMA checklist was used (Supplemental Table S1: PRISMA checklist) [16].

## 3. Results

From an initial pool of 160 records, 30 duplicates were removed, leaving 130 studies for title and abstract screening. Following this screening, 110 records were excluded due to being non-original articles (primarily reviews), addressing unrelated conditions, focusing on the surgical treatment of DDH, or being published in languages other than English. As a result, 32 full-text articles were assessed for eligibility. Most full-text articles were excluded due to a lack of appropriate outcome measures (*n* = 28). Four studies met the eligibility criteria (Figure 1).

All were prospective observational studies comparing motor development outcomes in children treated for DDH using abduction orthoses with healthy controls without DDH and without orthotic treatment. While one study was described by the authors as a prospective case–control, its methodology—prospective data collection and longitudinal follow-up of developmental milestones—more closely aligns with a comparative prospective cohort design [17]. Across all studies, outcomes were assessed based on the age of attainment of motor milestones, including unaided sitting, crawling, and walking (Table 1).

Collectively, these studies analyzed 952 children, with 335 treated for DDH using abduction orthoses (intervention group) and 617 healthy children without orthoses. In three studies, a Pavlik harness (*n* = 235) was used for intervention; in one study, a Koszla brace was used (*n* = 100). The intervention group consisted of 50 males (*n* = 50) and 285 females (*n* = 285; 85.07%). Sex distribution data for the control group were available in three of the four studies and included females (*n* = 311) and males (*n* = 206), indicating a predominance of female participants (60.10%). In the remaining study, sex-specific data for the control group were not reported (*n* = 100), and, therefore, the full distribution by sex remains unknown [20]. A chi-square test demonstrated a statistically significant difference in sex distribution between groups (*p* < 0.001). This imbalance may reflect the higher prevalence of DDH in females and should be considered when interpreting outcome differences between groups.

**Figure 2:**
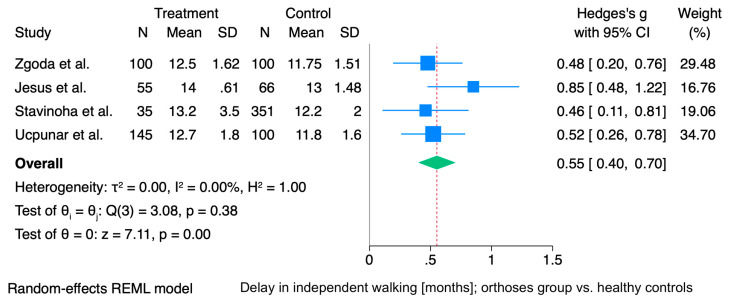
Forest plot showing the mean difference (MD) in age of independent walking (months) between the orthosis-treated and control groups. Horizontal lines represent 95% confidence intervals (CIs), with square size proportional to individual study weight. The diamond indicates the pooled MD with its corresponding 95% CI. For the study by Jesus et al., mean values and standard deviations were estimated using the method proposed by Hozo et al. [13,17,18,19,20].

The meta-analysis demonstrated a statistically significant mean difference of 0.55 months in the age of independent walking (95% CI: 0.40 to 0.70), indicating that children in the intervention group began walking approximately half a month later than those in the control group, and this is illustrated in Figure 2. It should be noted that the study by Jesus et al. had a disproportionately large influence on a slightly different orthotic treatment protocol and a high proportion of severe DDH cases (Graf III and IV), which may contribute to the observed heterogeneity among studies [17].

Meta-regression showed no significant association between the proportion of girls in the intervention group and age of independent walking (*p* = 0.465). Between-study variance remained unexplained (R^2^ = 0%), with low residual heterogeneity (I^2^ = 14.8%). Although the meta-regression suggested that longer orthosis treatment duration may be associated with a slight delay in the onset of independent walking (approximately 0.085 days per day of treatment), the relationship was not statistically significant (*p* = 0.45) and explained no heterogeneity across studies. We also did not identify a significant association between the age at orthosis initiation and the onset of independent walking. Each additional day of delay in starting treatment was associated with a non-significant (*p* = 0.76) delay in walking onset (approximately 0.063 days per day of delay in treatment initiation). Subgroup analysis showed no statistically significant difference between the Koszla (95% CI: 0.197–0.757) and Pavlik groups (95% CI: 0.386–0.788) in terms of independent walking (CI: 0.399–0.704, *p* = 0.531).

**Figure 3:**
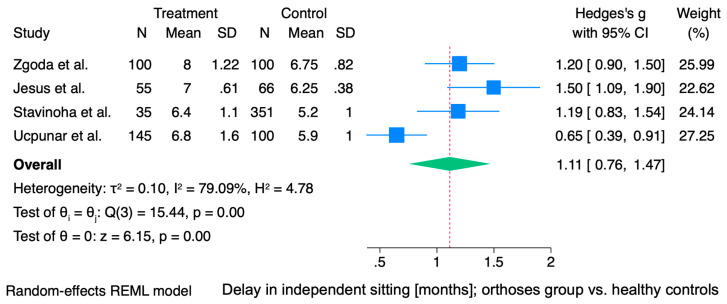
Forest plot illustrating the mean difference (MD) in age of independent sitting (months) between the orthosis-treated and control groups. Horizontal lines denote 95% confidence intervals (CIs), with square sizes proportional to the weight of each study. The diamond represents the pooled MD with its corresponding 95% CI. For the study by Jesus et al., mean values and standard deviations were estimated using the method described by Hozo et al. [13,17,18,19,20].

The meta-analysis demonstrated a statistically significant overall effect in favor of the control group regarding the age of unaided sitting. The pooled standardized mean difference was 1.11 months (95% CI: 0.76 to 1.47, *p* < 0.001), indicating that children in the intervention group sat independently at a noticeably later age compared to those in the control group. This substantial and potentially clinically relevant delay is illustrated in Figure 3. Between-study heterogeneity was considerable (I^2^ = 79.1%, τ^2^ = 0.10), suggesting meaningful variability across study results (Figure 3).

**Figure 4:**
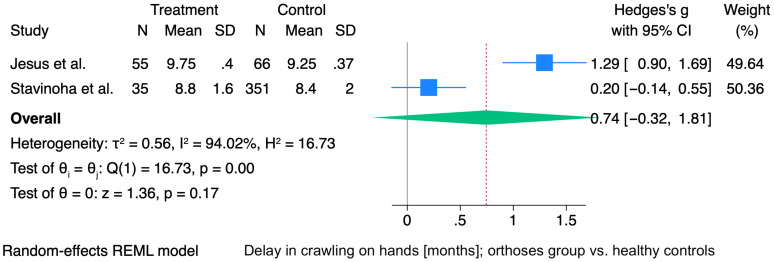
Forest plot showing the mean difference (MD) in age of crawling on hands (months) between the orthosis-treated and control groups. Horizontal lines indicate 95% confidence intervals (CIs), with square sizes proportional to individual study weights. The diamond represents the pooled MD with its corresponding 95% CI. For the study by Jesus et al., mean values and standard deviations were estimated using the method proposed by Hozo et al. [13,17], Stavinoha et al. [19].

The pooled analysis of two studies yielded a non-significant summary effect size for crawling on hands (Hedges’ g = 0.74; 95% CI: −0.32 to 1.81; *p* = 0.17). The study by Jesus et al. reported a large effect (g = 1.29), while the study by Stavinoha et al. showed a small, non-significant effect (g = 0.20). Heterogeneity was considerable (I^2^ = 94.0%; Q(1) = 16.73; *p* < 0.001), indicating substantial variation between studies (Figure 4).

**Figure 5:**
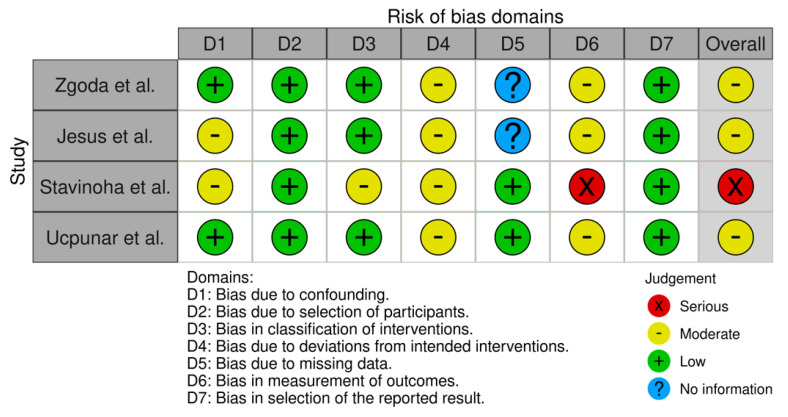
Traffic light plot summarizing the risk of bias assessment for included studies using the ROBINS-I tool. Each domain is color-coded to indicate low, moderate, or serious risk of bias, providing an overview of the methodological quality across studies [17,18,19,20].

**Figure 6:**
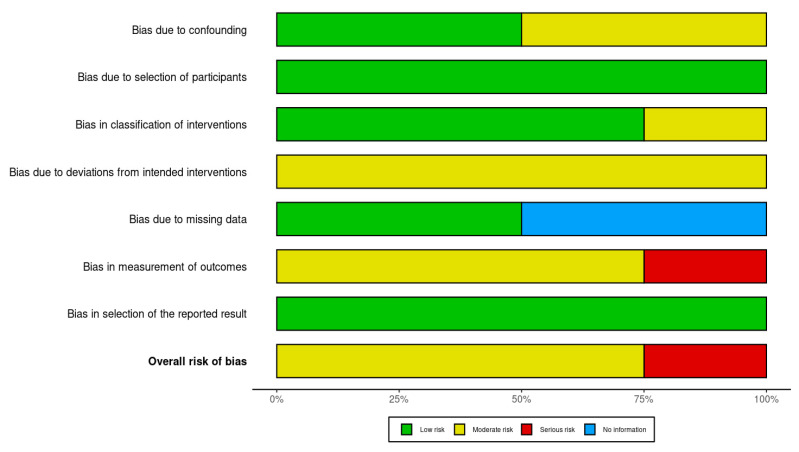
Summary plot of bias assessment for included studies using the ROBINS-I tool. The figure presents the overall distribution of risk of bias judgments across individual domains.

Two reviewers evaluated each study independently, covering key domains: selection, confounding, measurement, reporting, and missing data. Disagreements were resolved through discussion. The results are visualized using ROBVIS (Figure 5 and Figure 6). Due to the small number of included studies (*n* = 4), a formal assessment of publication bias using a funnel plot or Egger’s test was not performed.

## 4. Discussion

This is the first study to systematically review the impact of abduction orthosis treatment for DDH on the acquisition of key developmental milestones. The findings indicate very small, statistically detectable differences in the timing of independent sitting and walking in children treated with abduction orthoses compared with healthy peers; however, these differences are transient, minimal in magnitude, and fall within the broad range of normal motor development.

Several factors could hypothetically explain the observed delays in motor milestone acquisition. One possible contributor is the limited mobility imposed by abduction orthoses, which restrict spontaneous movement and reduce opportunities for balanced muscle strengthening and neuromotor practice. Unlike infants held in arms, those in orthoses demonstrate altered muscle activation patterns, with increased activity in the adductors, quadriceps, and hamstrings [21]. This compensatory recruitment may reflect postural adaptations to the fixed hip position, but it may not support the development of coordinated and functional movement patterns necessary for motor progression.

Moreover, abduction orthoses can potentially alter the infant’s postural alignment and weight distribution. Biomechanical analysis has shown that when placed in such devices, an infant’s shoulders are loaded with approximately 94% of their body mass, shifting the center of gravity and placing excessive demand on the upper body for stabilization [22]. This postural burden may compromise upper trunk mobility and diminish the ability to perform prone and transitional movements that are critical precursors to sitting. These mechanisms are consistent with findings from functional motor assessments. For instance, infants treated with the Pavlik harness have shown significantly lower scores on the Test of Infant Motor Performance following the application of the orthosis compared to pre-treatment baseline. This supports the hypothesis that orthotic treatment, although essential for hip joint stabilization, may transiently suppress early motor function through a combination of mechanical restriction, postural asymmetry, and altered neuromuscular engagement [23]. Additionally, the Pavlik harness is associated with neurological complications, most commonly femoral nerve palsy (4%) and, rarely, brachial plexus palsy. Although generally transient, these nerve injuries could possibly impair limb function and exacerbate existing muscle imbalances, further contributing to delayed motor development [24]. The biomechanical and neuromuscular mechanisms discussed should be regarded as hypothesis-generating interpretations, as they are not directly supported by the evidence derived from the included studies.

In addition to potential muscle weakening, the fixed hip position imposed by the orthosis may directly delay motor development—particularly in children with late treatment initiation or prolonged use due to more severe dysplasia. But there is no clinical evidence for this hypothesis so far. In such cases, the orthosis may physically restrict key movements, like rolling, crawling, or sitting, which typically emerge during the same developmental window as the immobilization period. According to the World Health Organization (WHO) Motor Development Study, sitting without support usually occurs between 3.8 and 9.2 months, while hands-and-knees crawling is achieved between 5.2 and 13.5 months (1st to 99th percentile) [25]. When orthotic treatment overlaps significantly with this developmental window, especially in prolonged treatment cases, it may interfere with opportunities for natural motor exploration and milestone acquisition.

These findings should be viewed in the broader context of DDH itself, which can delay motor development. Without treatment, DDH can lead to joint instability, pain, and structural problems that make it harder for children to stand and walk. The results of previous studies suggest that children with undiagnosed or late-treated DDH often have a delay in walking [9,26]. However, since all included studies enrolled children who were diagnosed and treated early, the likelihood that delays arose solely from untreated or late-treated DDH is low. Although orthotic treatment may cause a short-term delay in some milestones, it is still the most effective way to support proper hip development and prevent long-term consequences. The small delays seen in our study should be weighed against the serious risks of leaving DDH untreated.

This study has several important limitations that should be acknowledged. First, only four studies met the inclusion criteria, limiting the statistical power of the meta-analysis and reducing the ability to explore heterogeneity through subgroup or sensitivity analyses. Second, all included studies were observational and non-randomized, which increases the risk of selection bias and unmeasured confounding. Although the ROBINS-I tool was used to assess the risk of bias, residual bias cannot be excluded.

Third, the included studies varied in key aspects such as the type of orthosis used (e.g., Pavlik harness vs. Koszla brace), timing and duration of treatment, and methods of assessing motor milestones. This clinical and methodological heterogeneity may have influenced the pooled effect estimates. Considerable heterogeneity was observed in the analysis of independent sitting (I^2^ = 79.1%), suggesting substantial variability in study-level effects. This heterogeneity likely reflects differences in clinical management and study design, including variability in orthosis type and duration of treatment, baseline severity of DDH, and methods used to assess motor milestones.

Fourth, no included study compared children with DDH treated using orthoses to children with untreated DDH. Due to ethical concerns, such a comparison is not feasible in clinical research. As a result, it is not possible to fully separate the impact of the orthosis from the underlying effect of the condition itself. Therefore, while our results suggest a transient delay, the contribution of DDH itself cannot be ruled out, and the observed delays may reflect a combination of disease-related and treatment-related factors.

Fifth, a statistically significant difference in sex distribution between the intervention and control groups was observed, reflecting the higher prevalence of DDH in females. Multiple epidemiological studies have reported a higher risk of DDH in females (OR = 6.97, 95% CI: 5.18–9.39) [27], which is attributed to the greater susceptibility of female infants to maternal hormones such as relaxin, leading to increased joint laxity [28]. The higher prevalence of DDH among females observed in our study is consistent with findings from previous research [1]. Given that girls are more frequently affected by DDH and may show slightly earlier timing in achieving motor milestones compared to boys, this imbalance could potentially bias our results toward underestimating delays associated with orthotic treatment. However, recent studies often disregard differences between sexes in the age at which motor milestones are reached [25]. Nevertheless, the lack of a significant association in meta-regression should be interpreted with caution, as the limited number of included studies markedly reduces statistical power and does not preclude residual confounding related to sex distribution.

Lastly, in studies reporting outcomes as medians with interquartile ranges, mean values and standard deviations were estimated using the method proposed by Hozo et al. [13] This conversion may introduce imprecision, particularly in small samples or skewed distributions, and may have affected the accuracy of the pooled estimates [13].

Our risk of bias assessment revealed that the included studies demonstrated generally low to moderate risk across key domains. In one study, motor milestone data were collected through parental recall during routine follow-up visits and compared to external data from another study that used a different method—data collected by telephone—introducing potential inconsistencies in measurement and increasing the risk of bias [19]. This approach introduces a potential risk of recall and measurement bias, particularly for milestones achieved several months prior to data collection. Such misclassification may have influenced the precision of individual study estimates and, consequently, the pooled effect sizes, warranting cautious interpretation of the results.

To sum up, these findings support a cautious but reasonable confidence in the synthesized results. However, the small number of included studies (*n* = 4) and the inability to formally assess publication bias highlight the need for further high-quality research to validate and expand upon these conclusions. The lack of formal publication bias assessment should, therefore, be considered a structural limitation of the available evidence.

## 5. Conclusions

This systematic review and meta-analysis demonstrate that treatment of DDH with abduction orthoses is associated with a slight delay in the achievement of independent sitting and walking. These delays are likely multifactorial, reflecting the mechanical restrictions of the orthosis, postural adaptations, and—in some cases—neurological complications. However, given that untreated or late-treated DDH may lead to more severe and prolonged motor impairment, early orthotic intervention remains the standard of care. Clinicians should inform caregivers about the possibility of temporary developmental delays while reassuring them of the long-term benefits of timely and appropriate DDH treatment. These findings should be interpreted cautiously and primarily as hypothesis-generating, underscoring the need for further well-designed prospective studies with standardized outcome assessment. The milestone acquisition varies considerably among healthy infants, and such slight clinically marginal delays are unlikely to have any lasting impact on neuromotor outcomes. Therefore, orthotic treatment for DDH can be considered safe and non-invasive, effectively supporting hip development without causing clinically relevant delays in motor milestone achievement.

## Figures and Tables

**Table 1:** Identified study characteristics.

Study (Year)	Study Design	Sample Size (Intervention/Control)	Population Characteristics	Intervention (Part-Time or Full-Time)	Comparator	Outcome Measures
Zgoda et al. (2009) [18]	Prospective cohort	100/100	Sex Birthweight	DDH treated with a Koszla brace (not specified)	No DDH, without orthosis	Unaided sitting Unaided walking
Jesus et al. (2024) [17]	Author-described: a case–control study *	55/66	Sex Birth weight Gestational age Twin pregnancy Family history of DDH Presentation at birth Mode of delivery	DDH treated with Pavlik (not specified)	No DDH, without orthosis	Unaided sitting Unaided walking Crawling
Stavinoha et al. (2024) [19]	Prospective cohort	35/351	Sex	DDH treated with Pavlik (full-time and additional part-time wear)	Previously published cohort of healthy infants	Roll to supine Roll to prone Sit Crawl on stomach Crawl on hands Pull to stand Cruising Independent walking
Ucpunar et al. (2024) [20]	Prospective cohort	145/100	Sex ** Birthweight	DDH treated with Pavlik (full-time)	No DDH, without orthosis	Unaided sitting Unaided walking

* The study authors describe this as ‘prospective case–control’; however, data were collected prospectively and outcomes were observed over time, resembling a comparative cohort design. ** Data only for the intervention group.

## Data Availability

The STATA dataset is available on the Zenodo repository. These data include all extracted effect sizes, study descriptives, and descriptive statistics. These data have been assigned a digital identifier (https://doi.org/10.5281/zenodo.15586727). The STATA code used for data analysis is available upon request from the corresponding author.

## Supplementary Materials

The following supporting information can be downloaded at https://www.mdpi.com/article/10.3390/jcm15041595/s1, Table S1: PRISMA checklist [29].

## Abbreviations

The following abbreviations are used in this manuscript:

DDH	Developmental dysplasia of the hip
CI	confidence interval
SD	Standard deviation
MD	Mean difference
